# Dexmedetomidine Ameliorates Acute Stress-Induced Kidney Injury by Attenuating Oxidative Stress and Apoptosis through Inhibition of the ROS/JNK Signaling Pathway

**DOI:** 10.1155/2018/4035310

**Published:** 2018-09-03

**Authors:** Yongping Chen, Xiujing Feng, Xueyuan Hu, Jichen Sha, Bei Li, Huayun Zhang, Honggang Fan

**Affiliations:** College of Veterinary Medicine, Northeast Agricultural University, Harbin 150030, China

## Abstract

Acute stress induces tissue damage through excessive oxidative stress. Dexmedetomidine (DEX) reportedly has an antioxidant effect. However, protective roles and related potential molecular mechanisms of DEX against kidney injury induced by acute stress are unknown. Herein, rats were forced to swim 15 min followed by restraint stress for 3 h with/without DEX (30 *μ*g/kg). Successful model establishment was validated by an open-field test. Assessment of renal function (creatinine, urea nitrogen), histopathology, oxidative stress (malondialdehyde, glutathione, and superoxide dismutase), and apoptosis (transferase-mediated dUTP nick end labeling) was performed. Localization of apoptosis was determined by immunohistochemistry of cleaved caspase 3 protein. In addition, key proteins of the death receptor-mediated pathway, mitochondrial pathway, endoplasmic reticulum stress (ERS) pathway, and ROS/JNK signaling pathway were measured by Western blot. We found that DEX significantly improved renal dysfunction, ameliorated kidney injury, reduced oxidative stress, and alleviated apoptosis. DEX also inhibited the release of norepinephrine (NE), decreased the production of reactive oxygen species (ROS), and inhibited JNK phosphorylation. Additionally, DEX downregulated the expression of Bax, cytochrome C, cleaved caspase 9, and cleaved caspase 3 proteins in mitochondria-dependent pathways. In summary, DEX protects against acute stress-induced kidney injury in rats by reducing oxidative stress and apoptosis via inhibition of the ROS/JNK pathway.

## 1. Introduction

Stress can cause both physical and psychological disorders [1]. In recent years, stress has garnered wide concern because it causes diseases that pose great threats to public health [2]. At present, the most reported concern is that acute stress can affect the learning and working memory ability of people and animals [3, 4]. However, acute stress can also cause autoimmune disease [5], cardiovascular disease [6], acute myocardial infarction [7], liver damage [8], gastric ulcer [9], and brain damage [10]. The kidney is reportedly one of the most vulnerable organs in stress. Kidney injury can lead to renal insufficiency, hyperkalemia, water intoxication, fatal arrhythmia, and cerebral edema, which are often life-threatening [11, 12]. However, kidney injury induced by acute stress is rarely reported. Therefore, urgent study is needed regarding potential mechanisms and effective treatment targets of kidney injury caused by acute stress.

At present, the underlying molecular mechanism of acute stress-induced kidney injury is unclear. Previous studies have shown that injury of the liver [13] and hippocampus [14] caused by acute restraint stress is related to oxidative stress. As an inducer of apoptosis, oxidative stress can cause kidney damage [15]. In addition, apoptosis is involved in the occurrence and development of a variety of renal pathological injuries [16–18]. Thus, whether oxidative stress and apoptosis participate in the pathological process of kidney injury induced by acute stress should be further studied.

The ROS/JNK signaling pathway plays a crucial role in cell differentiation, apoptosis, and stress responses. When an organism receives a strong stimulus, this promotes excess ROS production [19]. Excessive ROS can break the balance between oxidative and antioxidant systems, thereby activating oxidative stress. Furthermore, ROS can activate JNK through apoptosis signal-regulating kinase 1 (ASK1) [20], Src kinase [21], glutathione s-transferase *π* (GST*π*) [22], mixed lineage kinase 3 (MLK3) [23], receptor-interacting protein (RIP) and tumor necrosis factor receptor-associated factor 2 (RAF2) complex [24], and mitogen-activated protein kinase phosphatase (MKPs) [25]. Norepinephrine (NE), a stress hormone, can induce apoptosis by activating the ROS/JNK signaling pathway [26]. In addition, studies have shown that ROS can mediate the JNK pathway to induce kidney injury [27]. Therefore, we speculated that inhibition of the ROS/JNK signaling pathway is an important protective mechanism for reducing kidney damage induced by acute stress.

Dexmedetomidine (DEX), a highly selective alpha 2 adrenal receptor (*α*_2_-AR) agonist, regulates the release of NE by acting on presynaptic membrane alpha 2 receptors [28]. Recent studies showed that DEX has a protective effect on kidney injury [29]. In addition, DEX has been shown to inhibit the release of proinflammatory cytokines [30, 31], attenuate apoptosis [32, 33], and reduce ROS production [34, 35]. However, protective roles of DEX during acute stress-induced kidney injury are unknown.

We hypothesized that DEX can improve acute stress-induced kidney injury. Thus, in the present study, we investigated regulatory effects of DEX on the ROS/JNK pathway and potential protective mechanisms against kidney injury induced by acute stress. The results provide a theoretical basis for future clinical research and the development of new antistress drugs.

## 2. Material and Methods

### 2.1. Animals and Housing

Adult male Wistar rats were obtained from the Second Affiliated Hospital of Harbin Medical University (Harbin, China). Rats weighed 180–220 g and were housed in a room that had a 12 h-12 h light/dark cycle (lights on from 6:00–18:00) with temperature 20 ± 2°C for one week to adapt to the environment. Rats were housed in groups (3 per cage) and had access to food and water ad libitum. All experimental procedures in this study met the requirements of the Animal Experimental Committee of Northeast Agricultural University, Harbin, China.

### 2.2. Animal Model

In the present study, an acute stress model was established by forced swimming for 15 min and restraint stress for 3 h. Eighteen rats were randomly divided into three groups as follows:

1. Control (C) group: the rats were not disturbed during acute stress.
2. Acute stress (AS) group: the rats were forced to swim in water (18–20°C) for 15 min (immediately removed when drowning occurred), then fixed on the fixture, exposing their limbs and head for 3 h.
3. Dexmedetomidine + acute stress (D + AS) group: the rats were treated with DEX (30 *μ*g/kg, i.p.) before acute stress.

### 2.3. Open-Field Test

After acute stress (AS), the open-field test was used to verify successful model establishment. The open-field box was a rectangular parallelepiped (100 cm × 100 cm × 40 cm) made of black wood without a lid. A camera was placed above the box to track and record rat performance. The bottom of the box was evenly divided into 25 squares. At the beginning of the test, rats were placed in the middle of the open-field box and the time was recorded. The behavior of rats within 3 min was observed and recorded, including immobility time, total distance, number of crossings, and number of rearing (frequency of both forelimbs being off the ground). Each rat was tested once. Open-field test results were analyzed and recorded using the Super Maze software (Shanghai, China).

### 2.4. Blood and Tissue Sample Collection

After the open-field test, blood samples were collected quickly by heart puncture and kept at room temperature for 30 min. The supernatant was collected by centrifugation at 3000 ×g for 10 min at 4°C and stored at −80°C until use.

Rat kidneys were rapidly separated, washed with 0.01 M phosphate-buffered saline, and placed in an ice tray. The left kidney was placed in 10% formalin solution and embedded in paraffin to observe pathological changes, while 200 mg of the right kidney was removed to immediately measure the concentration of reactive oxygen species (ROS). The remaining tissue was immediately frozen in liquid nitrogen and stored at −80°C for subsequent experiments.

### 2.5. Biochemical Analysis

The collected serum was measured for serum creatinine (CREA) and blood urea nitrogen (BUN) levels using a UniCel DxC800 Synchron (Beckman, USA). According to the instructions of the corresponding assay kit (Nanjing Jiancheng Bioengineering Institute, Nanjing, China), kidney tissue was prepared into a homogenate to detect concentrations of glutathione (GSH) and malondialdehyde (MDA), as well as activity of the antioxidant enzyme superoxide dismutase (SOD).

### 2.6. Histopathological Analysis

After fixation with 10% formalin solution, kidney tissue samples were dehydrated, seeded, dipped in wax, embedded, sectioned to about 4-5 *μ*m thickness, and stained with hematoxylin and eosin (H&E) (Wuhan Biotechnology Ltd. Co., Wuhan, China). Parameters of kidney injury were evaluated according to the following criteria: necrosis (presence of three or more cells with signs of coagulative necrosis, such as loss of cell boundaries with marked eosinophilia), vacuolar degeneration (presence of more than three cells with cytoplasmic vacuoles or bleb formation protruding into the lumen of tubules), neutrophil infiltration (presence of three or more neutrophils in the tubulointerstitial), and hemorrhage (presence of more than three red blood cells in the tubulointerstitial), as described by Chiazza et al. [36]. Six noncontinuous fields were evaluated from the cortex of each section (400x magnification). Rates of tubular necrosis and vacuolar degeneration were calculated based on percentages of corresponding tubules in each field. Furthermore, each field was divided equally into 100 grids and the percentage of the grid occupied by the hemorrhage point was the hemorrhage rate. The percentage of the grid occupied by neutrophil infiltration was the neutrophil infiltration rate. Necrosis, tubular degeneration, neutrophil infiltration, and hemorrhage scores were evaluated semiquantitatively as follows: 0, none; 1, <10%; 2, 10%–25%; 3, 25%–50%; and 4, >50% [37]. The sum of these changes represented the histopathological score for kidney injury. All sections were observed under a light microscope (TE2000, Nikon, Japan) and assessed by the same observer who was blinded to group assignment.

### 2.7. Immunohistochemistry Analysis

Sections were dewaxed, and then, antigen retrieval, primary antibody incubation, and labeling with horseradish enzyme were performed. Next, the color reaction was performed with a DNA coloring kit (Solarbio, Beijing, China) according to the manufacturer's instructions, and then, sections were counterstained with hematoxylin and made transparent with xylene. Finally, samples were observed with a DP73 type microscope (Olympus, Japan).

### 2.8. TUNEL Assay

Apoptosis was detected with a TUNEL Apoptosis Assay kit (Roche, Basel, Switzerland). The whole process was carried out according to the manufacturer's instructions, and samples were observed under a fluorescence microscope after antifluorescence quenching.

### 2.9. ELISA Assay

Concentrations of norepinephrine (NE), epinephrine, and corticosterone (CORT) in the serum of each group of rats were determined by enzyme-linked immunosorbent assay kit (Nanjing Jiancheng Bioengineering Institute, Nanjing, China) according to the manufacturer's instructions.

### 2.10. Western Blot

Frozen kidney tissues were cut into small pieces, lysed with RIPA lysis buffer (Beyotime Biotechnology, Shanghai, China) supplemented with phenylmethanesulfonyl fluoride (PMSF) (Beyotime Biotechnology, Shanghai, China), homogenized with a Tissue Prep instrument (Bio-Xplorer International Limited, Beijing, China), and then centrifuged at 12000 ×g for 10 min at 4°C to collect supernatant. Nuclear and plasma proteins were extracted using a nuclear-cytosol extraction kit (Beyotime Biotechnology, Shanghai, China). Protein concentrations were determined using an Enhanced BCA Protein Assay kit (Beyotime Biotechnology, Shanghai, China). Equal amounts of protein sample were loaded on SDS-PAGE gel, electrophoresed, and then transferred to PVDF membranes, which were blocked in 5% nonfat milk for 2 h at room temperature and then incubated overnight with primary antibodies in primary antibody dilution buffer (Leagene Biotechnology, Beijing, China) at 4°C. Primary antibodies and dilutions were as follows: 1 : 750 for caspase 8, lamin A/C, CHOP, GRP78/Bip, cleaved caspase 9, and cytochrome C (Wanlei Biotechnology, Shenyang, China); 1 : 1000 for Bax and cleaved caspase 3 (Cell Signaling Technology, USA); 1 : 1250 for TNF-*α*, XBP-1, and caspase 12; and 1 : 500 for Bcl-2 (Wanlei, Shenyang, China). After washing five times with Tris-buffered saline containing Tween (TBST), membranes were incubated with 1 : 6000 peroxidase-conjugated goat anti-rabbit IgG (ZSGB-BIO, Beijing, China) or goat anti-mouse secondary antibody at room temperature for 2 h and then washed with TBST. Immune-reactive protein bands were visualized using high-sig ECL Western blotting luminol/enhancer solution (Tanon Science & Technology Co., Shanghai, China), captured using the Amersham Imager 600 software (GE, USA), and quantified using the ImageJ software.

### 2.11. Statistical Analysis

Results were expressed as mean ± SEM (standard error means). Multiple datasets were compared with one-way analysis of variance (ANOVA) using the PASW Statistics 18 software (SPASS, IL, USA). Unpaired Student's *t*-test was used to compare the two sets of data. Graphs were made using GraphPad Prism 5 (San Diego, California). Quantitative analysis of integral optical density was performed using the Image-Pro Plus software (Media Cybernetics, Maryland, USA). *p* < 0.05 was considered statistically significant. Statistical differences were considered to be extremely significant when *p* < 0.01.

## 3. Results

### 3.1. Acute Stress Model Was Successfully Established

**Figure 1:**
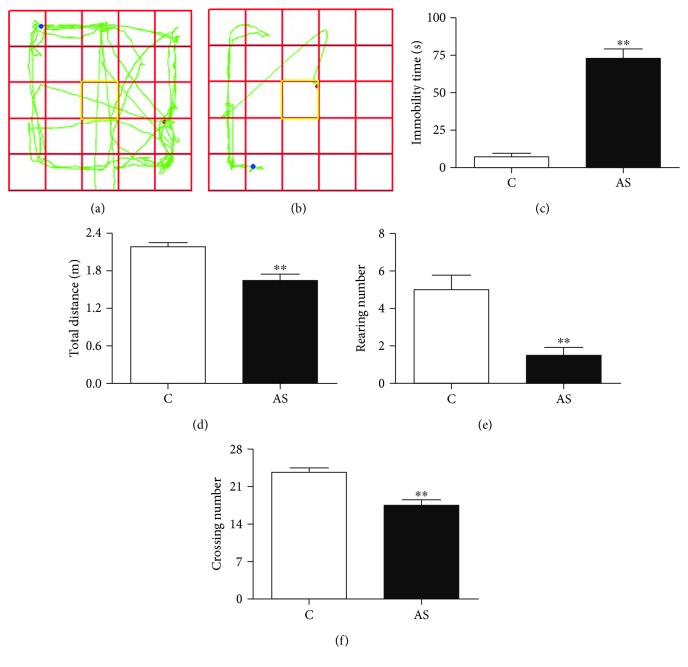
Results of the open-field test. The travel pathway of the open-field test: (a) C group and (b) AS group. The behavior of rats in the open-field test: (c) immobility time, (d) total distance, (e) rearing number, and (f) crossing number. Data are expressed as mean ± SEM (*n* = 6). ^∗∗^*p* < 0.01 versus C group.

To verify whether establishment of the acute stress model was successful, we measured the immobility time, total distance, and numbers of rearing and crossings by rats in the open-field test. As shown in Figures 1(a) and 1(b), acutely stressed rats exhibited more depression behavior in the open-field test. Immobility time in the AS group was significantly higher than that in the control group (Figure 1(c)). Conversely, significant reductions in total distance (Figure 1(d)), rearing number (Figure 1(e)), and crossing number (Figure 1(f)) were observed in the AS group compared with the control group. These results suggest that the acute stress model was successfully established.

### 3.2. DEX Improved Renal Dysfunction Induced by Acute Stress

**Figure 2:**
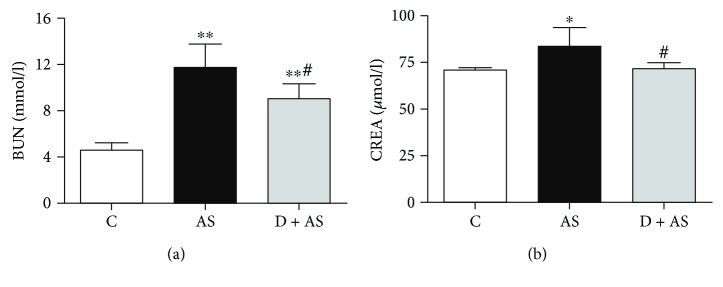
DEX improved renal dysfunction induced by acute stress. (a) The level of serum urea nitrogen (BUN) in rats. (b) The level of serum creatinine (CREA) in rats. Data are expressed as mean ± SEM (*n* = 6). ^∗^*p* < 0.05, ^∗∗^*p* < 0.01 versus C group. ^#^*p* < 0.05 versus AS group.

Levels of BUN and CREA in the AS group were remarkably higher than those in the control group. However, increased BUN and CREA levels were significantly reversed after treatment with DEX (Figures 2(a) and 2(b)).

### 3.3. DEX Ameliorated Renal Histopathological Injury Induced by Acute Stress in Rats

**Figure 3:**
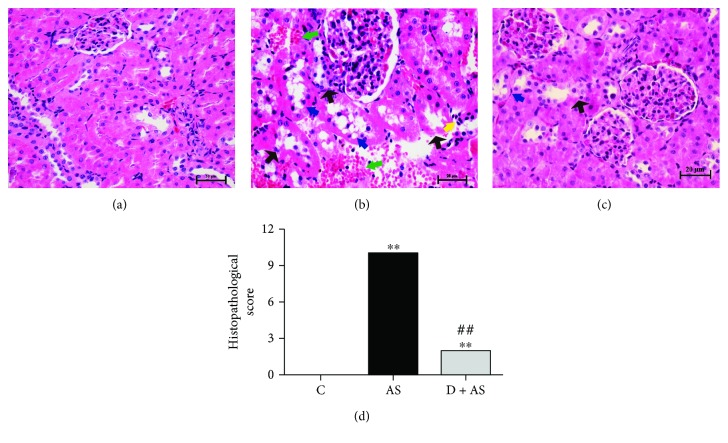
Protective effect of DEX on kidney injury induced by acute stress. Histopathological examination of the kidney (*n* = 6): (a) C group, (b) AS group, and (c) D + AS group. Black arrow indicates tubular necrosis; blue arrow indicates vacuolar degeneration; green arrow indicates hemorrhage; and yellow arrow indicates neutrophil infiltration. (d) Semiquantitative histopathological score of renal tissue injury. All histopathological sections were stained with hematoxylin and eosin for a magnification of 400. Data are expressed as mean ± SEM. ^∗∗^*p* < 0.01 versus C group. ^##^*p* < 0.01 versus AS group.

Histological examination was used to directly evaluate kidney injury. No abnormalities were observed in renal tubules, glomeruli, or renal interstitial in the control group (Figure 3(a), Table 1). In contrast, focal renal hemorrhage, neutrophil infiltration, focal tubular necrosis, and vacuolar degeneration of renal tubular epithelial cells were observed in the AS group (Figure 3(b), Table 1). However, after treatment with DEX, renal histopathological injury, such as neutrophil infiltration and hemorrhage, was not observed in renal tissue. Moreover, only a small amount of mild tubular necrosis was detected and only occasional vacuolar degeneration occurred in renal tubular epithelial cells (Figure 3(c), Table 1). Thus, treatment with DEX significantly reduced the severity of renal histopathological injury with respect to the AS group (Figure 3(d)).

### 3.4. DEX Ameliorated Acute Stress-Induced Oxidative Stress in the Kidney

**Figure 4:**
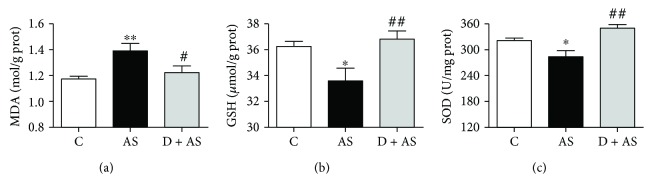
DEX reduced oxidative stress in acute stress. (a) The concentration of malondialdehyde (MDA) in rat kidney tissue. (b) The content of glutathione (GSH) in rat kidney tissue. (c) The activity of superoxide dismutase (SOD) in rat kidney tissue. Data are expressed as mean ± SEM (*n* = 6). ^∗^*p* < 0.05, ^∗∗^*p* < 0.01 versus C group. ^#^*p* < 0.05, ^##^*p* < 0.01 versus AS group.

The present study measured three important indicators of oxidative stress, including the level of MDA, content of GSH, and activity of SOD.  Figure 4(a) shows that acute stress induced a significant increase in MDA concentration in rat kidney tissue with respect to the control group. However, after treatment with DEX, these changes were significantly reduced. In addition, the concentration of GSH (Figure 4(b)) and activity of SOD (Figure 4(c)) in the AS group were significantly lower than those in the control group. Notably, concentrations of GSH and SOD in the D + AS group were significantly increased compared with those in the AS group.

### 3.5. DEX Alleviated Apoptosis of Rat Renal Tubular Cells Induced by Acute Stress

**Figure 5:**
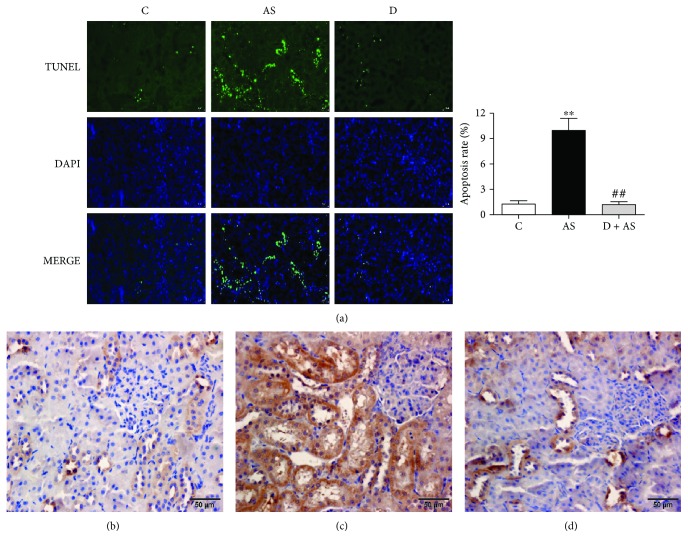
DEX improved the apoptosis of rat renal tubular cells induced by acute stress. (a) The apoptosis of renal tissue was measured by the TUNEL Apoptosis kit, in which green cells represent TUNEL-positive apoptotic cells. TUNEL (green) detects apoptosis, DAPI (blue) locates the nucleus, and MERGE is the combination of TUNEL and DAPI. All fluorescence images were observed with a fluorescence microscope at a magnification of 200. Quantitative results of TUNEL analysis. Immunohistochemistry of cleaved caspase 3 protein: (b) C group, (c) AS group, and (d) D + AS group; bars = 50 *μ*m. Data are expressed as mean ± SEM (*n* = 6). ^∗∗^*p* < 0.01 versus C group. ^##^*p* < 0.01 versus AS group.

The TUNEL assay was used to assess the apoptosis of the kidney. As shown in Figure 5(a), the control group had very few apoptotic cells. In contrast, apoptotic cells in the AS group were significantly increased, mainly in renal tubules, and the rate of apoptosis increased. Treatment with DEX obviously reduced numbers of apoptotic cells in rats.

Cleaved caspase 3 is the most important terminal cleavage enzyme and is considered the ultimate common pathway member for different apoptotic cascades. Therefore, the site of kidney injury was determined by measuring localization of cleaved caspase 3 in kidney tissue. As shown in Figures 5(b) and 5(c), cleaved caspase 3 was primarily expressed in renal tubules. However, DEX significantly inhibited the expression of cleaved caspase 3 and reduced apoptosis of renal tubular cells (Figure 5(d)).

### 3.6. DEX Protected against Acute Stress-Induced Kidney Injury by Inhibiting the Internal Mitochondrial Apoptotic Pathway

**Figure 6:**
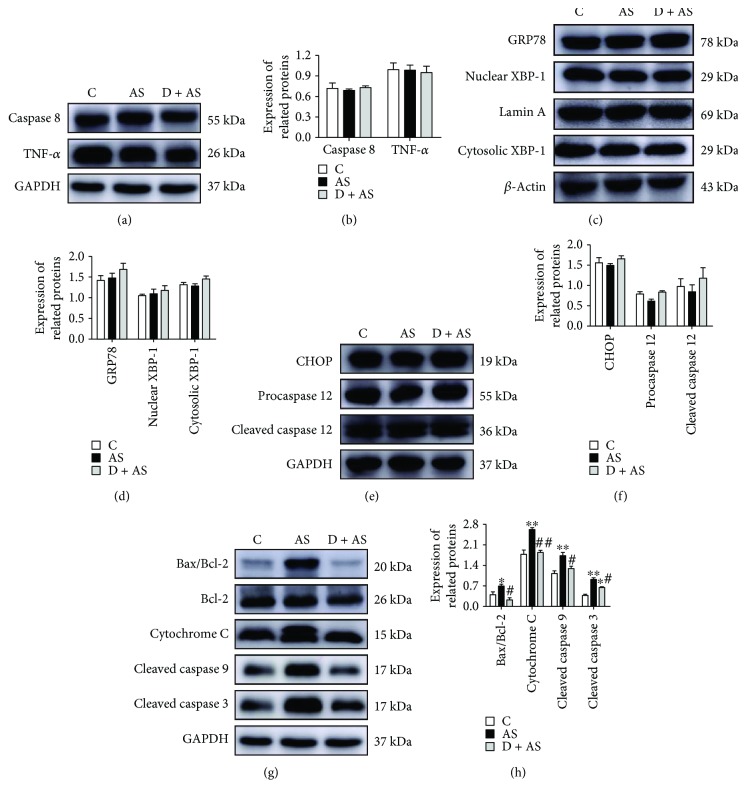
DEX protected against acute injury-induced kidney injury primarily by improving the internal mitochondrial apoptotic pathway. Expression (a) and quantitative analysis (b) of key proteins in the death receptor pathway. Expression (c) and quantitative analysis (d) of GRP78, cytoplasm, and nucleus XBP-1 in the kidney. Expression (e) and quantitative analysis (f) of crucial proteins in the endoplasmic reticulum stress apoptosis pathway. Expression (g) and quantitative analysis (h) of vital proteins in the internal mitochondrial pathway. Data are expressed as mean ± SEM (*n* = 4). ^∗^*p* < 0.05, ^∗∗^*p* < 0.01 versus C group. ^#^*p* < 0.05, ^##^*p* < 0.01 versus AS group.

To investigate which apoptotic pathway is primarily activated by acute stress and whether DEX exerts protective effects on this pathway, key proteins in classical apoptotic pathways including the death receptor pathway, mitochondrial apoptotic pathway, and endoplasmic reticulum stress (ERS) pathway were investigated. Expression of key proteins in the death receptor-mediated apoptosis pathway (caspase 8 and TNF-*α*) in renal tissue of the AS group was not statistically different compared with that of the control and D + AS groups (Figures 6(a) and 6(b)). Cytoplasmic and nuclear expressions of XBP-1 protein in all three groups of rats were identical, with no statistical differences (Figures 6(c) and 6(d)). There was no significant difference in expression of key proteins in the ERS pathway (GRP78, CHOP, procaspase 12, and cleaved caspase 12) in the three groups (Figures 6(e) and 6(f)). However, protein expression of Bax/Bcl-2, cytochrome C, cleaved caspase 9, and cleaved caspase 3 in renal tissue of the AS group was significantly higher than that of control rats (Figures 6(g) and 6(h)). Thus, our results showed that the mitochondrial apoptotic pathway was activated by acute stress. Moreover, increased expression of Bax, cytochrome C, cleaved caspase 9, and cleaved caspase 3 protein was markedly reversed by DEX.

### 3.7. DEX Inhibited Norepinephrine Release in Acute Stress

**Figure 7:**
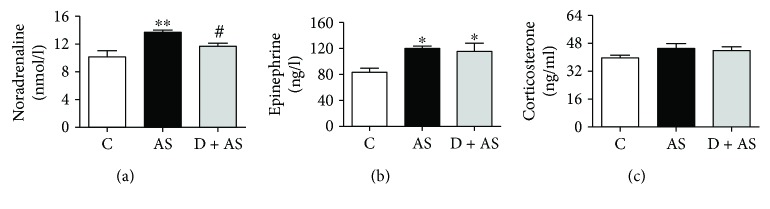
DEX inhibited norepinephrine release in acute stress. (a) The level of NE in rats. (b) The level of epinephrine in rats. (c) The level of CORT in rats. The data is expressed as mean ± SEM (*n* = 6). ^∗^*p* < 0.05, ^∗∗^*p* < 0.01 versus C group. ^#^*p* < 0.05 versus AS group.

The main neuroendocrine response of the body during stress is excitation of the hypothalamic-pituitary-adrenal cortex (HPA) axis and blue-spot-sympathetic-adrenal medulla (LC/NE) axis, which significantly increases the concentration of corticosterone (CORT), norepinephrine (NE), and epinephrine in the blood. We speculated that the protective effect of DEX against acute stress-induced kidney injury may be related to CORT, NE, and epinephrine. Therefore, levels of NE, epinephrine, and CORT were detected in the present study. As shown in Figures 7(a) and 7(b), levels of NE and epinephrine were significantly increased in the AS group after acute stress compared with the control group. Interestingly, only the level of NE was significantly reduced after DEX treatment. In addition, although the level of CORT in the AS group was higher than that in the control group, the difference was not significant (Figure 7(c)). The results of this study indicated that DEX mainly inhibits the release of NE in acute stress.

### 3.8. DEX Protected against Acute Stress-Induced Kidney Injury by Mediating the ROS/JNK Pathway

**Figure 8:**
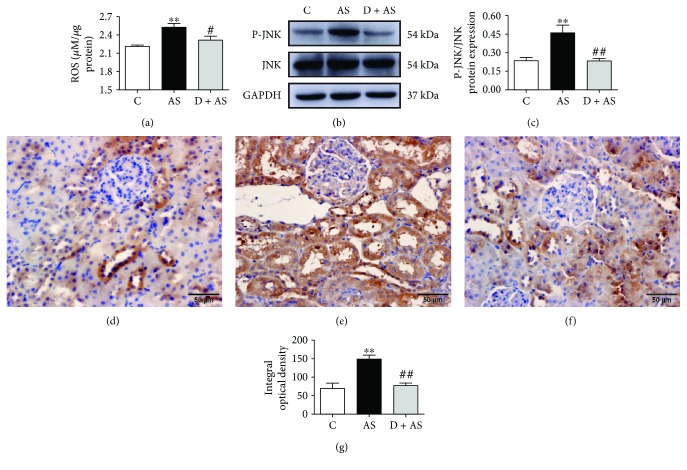
DEX protected against acute stress-induced kidney injury by mediating the ROS/JNK pathway. (a) The content of ROS in kidney tissue. (b) Protein of JNK and P-JNK in renal tissue. (c) Quantitative analysis of protein expression of P-JNK/JNK in renal tissue. Immunohistochemistry of P-JNK protein: (d) C group, (e) AS group, and (f) D + AS group; scale bars = 50 *μ*m. (g) Quantitative analysis of P-JNK in renal tissues. Data are expressed as mean ± SEM (*n* = 6). ^∗∗^*p* < 0.01 versus C group. ^#^*p* < 0.05, ^##^*p* < 0.01 versus AS group.

Compared with the control group, levels of ROS in kidneys of the AS group were significantly increased. However, DEX obviously inhibited ROS production and played a protective role (Figure 8(a)). After acute stress, the protein level of phosphorylated JNK (P-JNK) in the AS group was significantly increased (Figures 8(b), 8(c), and 8(g)) and mainly expressed in renal tubules (Figures 8(d)–8(f)). Treatment with DEX significantly inhibited P-JNK expression.

## 4. Discussion

Acute stress occurs frequently in daily life. An increasing number of studies have shown that acute stress can induce multiple organ damage [13, 14]. To study whether acute stress can induce kidney injury, an acute stress model was established by forced swimming for 15 min and restraint stress for 3 h based on previous studies [38–42]. Results of the study showed that the levels of CREA and BUN were significantly increased after acute stress, indicating impairment of renal function. In addition, histopathological examination provided further evidence for kidney injury caused by acute stress. However, such injuries were obviously attenuated by DEX. Therefore, DEX could be an effective drug to inhibit kidney injury induced by acute stress. To the best of our knowledge, the present study is the first to investigate the effects of DEX on antistress and protection mechanisms against acute stress-induced kidney injury.

When the organism is in a state of stress, it produces excessive oxygen free radicals, which destroy the balance between oxidation and antioxidant system [19, 43]. Previous studies have found that oxidative stress can lead to kidney damage [44, 45]. MDA, which indirectly reflects the severity of damage induced by free radicals, is an important biomarker of oxidative damage [46], whereas SOD and GSH are important antioxidants [47]. The current results indicated that acute stress impaired the function of the antioxidant defense system by increasing the level of MDA and decreasing the enzyme activity of SOD and GSH. Thus, oxidative stress induced by acute stress may be important to the pathogenesis of kidney injury. However, treatment with DEX ameliorated those revisions. Consistent with the findings of previous studies, DEX has an antioxidative stress effect [34, 35], which may elicit a protective mechanism of DEX against kidney injury induced by acute stress.

Apoptosis, a physiological mechanism of organisms, plays an extremely important role in maintaining the stability of internal environments. However, the excessive apoptosis has adverse effects on the body. According to reports, apoptosis induces damage to a variety of tissues, including those of the kidney [48, 49]. In the present study, TUNEL results showed that apoptosis was involved in the process of kidney injury induced by acute stress. Recently, studies have demonstrated the antiapoptotic function of DEX in tissues such as those composed of neurons [50] and cardiomyocytes [51] and those of the hippocampus [52]. In the current study, DEX alleviated acute stress-induced apoptosis by reducing the number of apoptotic cells in stress model rats. Thus, the antiapoptotic effect of DEX may elicit a protective mechanism against kidney injury induced by acute stress.

The kidney is composed of tubules, renal vesicles, and glomeruli. Studies have reported that kidney injury is mainly caused by glomerular or tubular apoptosis [53, 54]. However, whether DEX alleviates apoptosis induced by acute stress in tubular cells or glomerular cells is unclear. Immunohistochemistry permits localization and quantification of antigens in tissue by specific antibodies [55]. The final common pathway of different apoptosis cascade reactions is activation of caspase 3 expression. Thus, immunohistochemistry of cleaved caspase 3 was examined as an indicator of tubular apoptosis. After treatment with DEX, the effect of acute stress induction was significantly improved. As such, the current study demonstrated that DEX can reduce acute stress-induced apoptosis of renal tubular cells.

So far, the apoptotic pathway is known to include the death receptor-mediated pathway, the mitochondrial pathway, and the endoplasmic reticulum stress (ERS) pathway. However, the specific apoptotic pathway activated by acute stress is unknown. Tumor necrosis factor (TNF) reportedly leads to kidney injury [56]. Additionally, TNF-*α* induces apoptosis by activating caspase 8 [57]. A recent study showed that oxidative stress can activate caspase 8, which is the direct target of death receptors that mediate apoptosis [58]. Interestingly, our results indicated that acute stress did not activate the death receptor pathway, a result that may arise from the use of different models.

Oxidative stress activates the ERS-related apoptosis pathway [59]. In addition, this previous study showed that ERS was involved in the pathological process of kidney injury [60]. However, our results show that there is no significant increase in GRP78, CHOP, caspase 12, or nuclear XBP-1 protein expression in the kidney after acute stress; cytoplasmic protein expression of XBP-1 was not decreased. This may be related to the duration or intensity of stress. However, further research is necessary.

A recent study showed that oxidative stress induces mitochondrial apoptosis, which in turn leads to kidney injury [61]. To determine whether acute stress induced kidney injury by activating the mitochondrial apoptosis pathway, follow-up studies were performed. The ratio of proapoptotic protein Bax and antiapoptotic protein Bcl-2 is a key factor in regulating apoptosis. When Bax is overexpressed and Bax-Bax homodimers are formed, apoptosis is promoted. Bax oligomers are inserted into the mitochondrial membrane to increase mitochondrial permeability and open mitochondrial permeability transition pore (MPTP). Concomitantly, cytochrome C in mitochondria is released into the cytoplasm, whereby it participates in the formation of apoptotic bodies. Caspase 9 combines with apoptotic bodies to activate caspase 3 and induce apoptosis [62]. Our findings revealed that acute stress induced kidney injury mainly by activating the mitochondrial apoptosis pathway. However, increased protein expression of Bax, cytochrome C, cleaved caspase 9, and cleaved caspase 3 was reversed after treatment with DEX. Our results demonstrated that DEX can relieve acute stress-induced kidney injury by inhibiting mitochondrial apoptosis pathways.

The sympathetic-adrenal medulla (SAM) system and hypothalamus pituitary adrenal (HPA) axis are two important neuroendocrine systems for stress responses [63]. The SAM system, which secretes NE and epinephrine, is one of the earliest systems involved in the stress response [64], whereas the HPA axis is stimulated to release CORT during stress [65]. Surprisingly, our findings showed that levels of NE and epinephrine in blood significantly increased after acute stress, while the level of CORT was not significantly increased. This may be related to rats being in the third phase of stress (exhaustion period). Importantly, NE release was reduced after treatment with DEX. It has been reported that NE produced by SAM undergoes self-oxidation in the bloodstream to produce oxygen free radicals [66]. Moreover, previous studies have shown that NE can induce ROS production [67, 68]. Therefore, excessive amounts of NE in acute stress may self-oxidize, producing large amounts of ROS, causing oxidative stress and thereby inducing kidney injury. Thus, the protective effect of DEX on acute stress-induced renal injury may be related to inhibition of NE release. However, the specific mechanism needs further research.

During acute stress, the organism produces a lot of ROS, which cause oxidative stress. As a signal molecule, ROS can activate JNK through ASK1, Src, GST*π*, and MLK3 pathways. JNK, a stress-activated protein kinase (SAPK), can modulate Bcl-2 family proteins, activate Bcl-2 proapoptotic proteins, and inhibit antiapoptotic protein activity. Moreover, studies have reported that JNK induces apoptosis [69]. In addition, the ROS/JNK signaling pathway can mediate mitochondrial internal apoptosis. ROS can also mediate the JNK pathway to induce kidney injury. Our study found that DEX reduced ROS production by enhancing the antioxidant system and inhibiting JNK phosphorylation. Therefore, inhibition of the ROS/JNK pathway may be a potential protective mechanism for DEX to ameliorate acute stress-induced renal injury.

## 5. Conclusion

In conclusion, the present study demonstrated that DEX protected against acute stress-induced renal tubular injury, which may be effective by regulating NE release, strengthening the antioxidative stress system, reducing ROS production, and inhibiting JNK phosphorylation, thereby downregulating expression of key proteins in the mitochondria-dependent pathway. This study provides a theoretical basis for future development of new antistress drugs and guidance for the clinical application of DEX as an antistress agent.

## Figures and Tables

**Table 1:** The rate of histopathological lesion in different groups.

Histopathological lesion	C	Experimental groups	
		AS	D + AS
Tubular necrosis	0.00	70.67 ± 0.72^∗∗^	5.47 ± 0.12^∗∗^^,##^
Vacuolar degeneration	0.00	31.83 ± 0.72^∗∗^	2.00 ± 0.24^∗∗^^,##^
Neutrophil infiltration	0.00	1.59 ± 0.038^∗∗^	0.00^##^
Hemorrhage	0.00	18.45 ± 0.73^∗∗^	0.00^##^

Data are expressed as mean ± SEM. ^∗∗^*p* < 0.01 versus the C group. ^##^*p* < 0.01 versus the AS group.

## Data Availability

The data used to support the findings of this study are included within the article.
